# Athletics and Motherhood: An American View

**Published:** 1912-11-30

**Authors:** 


### MATERNITY AND MOTHERING.

(Inquiries and Correspondence are invited.)

### Athletics and Motherhood: An American View.

When the tendency for women and girls to take part in outdoor sport first began to get a real root in this country—say thirty to thirty-five years ago —there were distinguished obstetricians of the day who shook their heads over the innovation and prophesied dreadful things about the future fate of the athletic girl when the part of maternity should fall to her lot. These prognostications fell in so aptly with the prejudices of the older generation of the mothers and grandmothers of the day that the latter were strongly fortified in their aversion to the "modern craze for sport"; but they did not avail to deter healthy girlhood from participation in athletics, which, as we now see, are more popular and more widespread than ever. It is, however, time that some serious attempt should be made to decide scientifically whether the function of maternity is helped or hindered in those who have indulged in outdoor athletic pastimes. Such an inquiry should distinguish, if possible, between the effects of moderate reasonable indulgence and those of irrational excess. It should also investigate not only the effects of sport upon the pelvic bones, but also those upon all the factors which are involved in labour, such as the muscular tone of the uterus, the resistance of the pelvic floor, perineum, and ligaments; and also upon lactation, menstruation, and the whole of the apparatus which subserves the

Single issue lest the generations fail.

A New York medical woman, Dr. Angenette Parry, has conducted an inquiry somewhat on these lines; and her conclusions are published in the *American Journal of Obstetrics and Gynæcology*. It is to be noted that Dr. Parry's investigation does not profess to be anything more than a preliminary contribution to the problem she is attacking; but it is to be hoped that some day she will publish a full account of her researches and the data upon which her conclusions have been based. In this country it is probable that the competitive element in athletics amongst girls' and women's colleges is not pushed to the same excess as in America; so that some of Dr. Parry's conclusions may need modification in their application to conditions here in Britain.

### Menstruation.

To begin with, the relation of physical outdoor exercise and games to the function of menstruation was reviewed. Here, perhaps, it should be mentioned at once that Dr. Parry circularised a very large number of obstetrical and gynæcological experts, to whom a long series of specific questions were addressed. Her analysis of the answers received constitutes the main part of her paper; and she found her correspondents by no means unanimous in their opinions. So that the views expressed are those of a majority which may not always be a large one, though as a matter of fact in regard to many points the minority was not of very considerable dimensions.

Moderate athletic exercises, all are agreed, are beneficial to the general health of girls. Most schools, colleges, and gymnasiums excuse their girls from such exercise for one to three days at the onset of menstruation. Some authorities would vigorously interdict all exercise during menstruation; others, on the other hand, allow moderate exercise to normal healthy girls during, as well as between, the periods. Those who follow the latter plan point to the case of circus riders and other acrobatic performers who cannot be thus excused and seem to suffer extraordinarily few ill-effects in consequence. Without laying down any hard-and-fast rule, it may be said that there are many girls and women for whom moderate athletics during menstruation are permissible; while there are others for whom a rest of two or three days is imperative. The individual case must be studied, and the greatest care taken that athletics should be abandoned at this time if it is apparent that they interfere with the normal evolution of the menstrual function.

### Parturition.

The most important section, perhaps, of the whole subject is the effect which athletic exercises influence upon the course of childbirth; and on this matter opinions vary very considerably. Rather more than half of those who were asked to express an opinion on this point believe that the well-developed athletic woman passes through this ordeal more successfully than do those who have followed sedentary lives or been engaged in manual labour. But a considerable minority do not subscribe unreservedly to that view. Although it may be granted that the strong, healthy, open-air girl has a far greater chance of bearing a large, robust child, and that if complications arise she has a greater reserve of health and strength to draw upon, it does not follow that the actual process of labour is shorter or easier. Greater muscular development of the uterine and abdominal walls may exist; but its effect on the actual process of labour may be nullified by a greater resisting power of the pelvic floor and perineum. Some authors hold that the latter element counterbalances the former, and the present writer, as far as his own experience goes, is inclined to favour this contention. Perineal tears and lacerations of the cervix and vagina would seem to be distinctly commoner in athletic than in non-athletic women; and the whole process of childbirth may be longer as well as more exacting. Here, again, dogmatic assertions are inadvisable; statistical evidence one way or the other seems impossible to obtain, and we must be content with recording impressions, which may turn out some day to be erroneous.

As regards lactation, and metabolism in general after childbirth, the athletic woman certainly scores over the less developed type; of this there can be little doubt. Whether pelvic abnormalities and contractions are more or less frequent among the athletic there seems to be no reliable evidence. Most likely, one would suppose, they would be less frequent; but it is better not to prejudge the question pending a thorough investigation.

### Uterine Displacements.

The possibility of acquiring or accentuating displacements of the uterus through excessive indulgence in violent exercise is another topic which has much divided the medical profession. Five out of twenty gynæcologists believe that such displacements can be caused by injudicious violent exertion, especially if it is indulged in during the menstrual period. Several others specify that if there exists already a slight retroversion, athletic exercise tends to aggravate it. Many physicians hold that pelvic trouble of this nature is only likely to arise when a girl who is quite out of training, with soft relaxed muscles, suddenly takes part in some severe competitive game. Feats which have been performed with perfect safety when in good training may thus result in harm if attempted when quite untrained: an observation so completely in harmony with what is known about the effect of athletic exercises upon the heart that we may fairly accept it as accurate. Some directors of gymnastic and other institutes for physical training believe firmly that athletic work begun early and carried out regularly is actually a prophylactic against uterine abnormalities and that it may even remedy existing displacements.

Dr. Parry concludes with a survey of the subject which commends itself to attention on account of its moderate tone and sensible expression of kindly wisdom. She would allow little girls to lead the same out-of-door unconventional life as their small brothers do. Swimming, running, ball-games, and similar sports should be permitted within reason up to the age of puberty. Athletic games after that epoch should be supervised by an experienced physical director, and each girl who partakes of them should have been carefully examined and passed as sound by a physician conversant with the scope of athletics and the effects of specified forms of exercise. By the time a girl has left school, whether to carry on her education at a college or to return to her home, she should have the advantage of a thorough grounding in the main physiological facts of her general and her special pelvic development; she should know that penalties will in the end be incurred by recklessness and by exercise too violent or too stimulating for her individual organisation. She should be taught to heed the warnings of Nature, and especially she should have a clear idea of what she can and what she cannot and must not do during her menstrual period. Great exertion, particularly of a competitive kind, can be permitted only to a very few at this time; such cases are exceptional, and the majority will find that moderate exercise is the most they can safely take, while others will find it best to rest altogether.

The whole subject of competitive inter-collegiate athletic contests for girls is really determined by a consideration of these conclusions. Since it is manifestly impossible to arrange the fixtures between teams in such a way as to clash with the menstrual period of no single member of them, the entire system of such strenuous and exciting competitions stands condemned. The nervous strain involved in them is very great, and there is always the risk that the intoxication of athletic success will result in mental as well as physical over-strain and breakdown. Enough has been said to show that the regulation of athletics for girls and young women is a matter of the greatest moment; and it is to be hoped that the matter will not be allowed to rest, but that trustworthy data will be secured from which scientifically exact conclusions can be drawn. At present all opinions are tentative and tinged by the personal predilections of those who pronounce them. What seems to one teacher reasonable and moderate exercise may to another seem violent and unreasonable. What is a fitting and healthful amount of exertion for one individual may be senseless over-exertion to another. And, finally, what can be safely undertaken by a woman in good condition may be most deleterious to the same individual when "untrained" or unfit.

